# Performance of a Protected Wireless Sensor Network in a Fire. Analysis of Fire Spread and Data Transmission

**DOI:** 10.3390/s90805878

**Published:** 2009-07-24

**Authors:** Thierry Antoine-Santoni, Jean-François Santucci, Emmanuelle de Gentili, Xavier Silvani, Frederic Morandini

**Affiliations:** University of Corsica/UMR CNRS SPE, Quartier Grossetti 20250 Corte, France; E-Mails: santucci@univ-corse.fr (J.-F.S.); gentili@univ-corse.fr (E.G.); silvani@univ-corse.fr (X.S.); morandin@univ-corse.fr (F.M.)

**Keywords:** wireless sensor network, thermal insulation, natural fire, fire spread

## Abstract

The paper deals with a Wireless Sensor Network (WSN) as a reliable solution for capturing the kinematics of a fire front spreading over a fuel bed. To provide reliable information in fire studies and support fire fighting strategies, a Wireless Sensor Network must be able to perform three sequential actions: 1) sensing thermal data in the open as the gas temperature; 2) detecting a fire i.e., the spatial position of a flame; 3) tracking the fire spread during its spatial and temporal evolution. One of the great challenges in performing fire front tracking with a WSN is to avoid the destruction of motes by the fire. This paper therefore shows the performance of Wireless Sensor Network when the motes are protected with a thermal insulation dedicated to track a fire spreading across vegetative fuels on a field scale. The resulting experimental WSN is then used in series of wildfire experiments performed in the open in vegetation areas ranging in size from 50 to 1,000 m^2^.

## 1. Introduction

The Mediterranean area is a region experiencing strong ecological variations due to global climate change: wildland and forest fires represent there an important cause of natural hazards and disasters for assets and human lives every year. Early detection and accurate tracking of the fire front propagation are key points in fire fighting strategies to minimize the damage and possible casualties. According to fire managers, the delay in detecting a fire ignition in the open is considered as one of the main factors increasing the response time of the first fire fighting actions. The resulting size of the fire to be managed as the final burning area strongly depends on this response time of the first fire fighting action (FFFA). According to the Prométhée database—the inventory of fires in the Mediterranean region in the South of France since 1973—only the alerts given by operational services (firemen, forest patrols) lead to a FFFA response time lower than 15 minutes due to the quality of information on the accurate location of the fire: this database reports that 66% of all fire detections are performed by civilians. Furthermore, reference [1] mentions that the increase in the number of fire lookout towers and patrols of forest managers significantly decreases the final size of burned areas. In both cases, the decrease of the burned vegetation area can be attrbuted to short FFFA response times. Moreover, in standard models for fire fight and forest manager, the fire power. i.e., the amount of heat release by time and length units of fire front is linearly dependant on the fire rate of spread (ROS). This means that the accurate tracking of the fire front spread leads to information of first rate importance in terms of fire fighting.

The present study aims to illustrate that a device for detecting the presence of a fire and for tracking its space and time evolution can be designed from a Wireless Sensor Network. Indeed, a Wireless Sensor Network (WSN) meets the technical needs to provide accurate environmental data and robustness for use in a fire environment. Advances in Micro-Electro-Mechanical-System (MEMS) based sensor devices and miniaturization of processors and radios as sensor packages have led to the emergence of sensor networks. A sensor node must support the following abilities: computing, communicating and sensing. The sensor sends collected data, usually via a radio transmitter, to a command center (sink or base station) either directly or through a data concentration center (gateway). In a sensor network, different functionalities can be associated with the sensor nodes. The sensor nodes are usually scattered in a sensor field. Each of these scattered sensor nodes is able to collect data and route this data back to the sink/base station using a multihop infrastructureless architecture. The sink may also communicate with the task manager node via Internet or satellite. The design of the sensor network is influenced by many factors, including fault tolerance, scalability, production costs, operating environment, sensor network topology, hardware constraints, transmission media, and power consumption.

Our contribution in this framework was to design a new tool for monitoring wildland and forest fires using a WSN. We show in this paper that a Wireless Sensor Network can perform the following sequence of tasks:

1. sensing thermal data in the open: the system must be able to sense accurate critical environmental data (the air hygrometry for instance) for zoning a risk of fire ignition;
2. detecting a fire ignition: the sensing of thermal data must be sufficiently adapted and accurate to the detection of a fire environment as a temperature elevation due to the presence of a gaseous flame;
3. tracking the fire spread during its spatial and temporal evolution.

In the following, we present a short description of the state-of-art of existing WSN systems in fire monitoring in order to underline the originality of the present contribution.

### 1.1. Overview of the Existing Literaure Involving WSNs

WSNs may potentially provide a solution to the previously mentioned requirements (1), (2) and (3). Recent advances in WSNs support our belief that they constitute a promising framework for building near real-time forest fire detection systems. Currently, sensing modules can sense a variety of phenomena, including gas temperature and relative moisture content, two essential parameters in fire detection. Sensor nodes can operate for months on a pair of AA batteries to provide constant monitoring during the fire season. Moreover, recent protocols make sensor nodes able to organize themselves into a self configuring network, thus removing the overhead of manual setup.

The feasibility of using wireless sensor networks for forest fire monitoring is illustrated in [2,3]. Experimental results from two controlled fires in San Francisco (California, USA) were reported. The system is composed of 10 GPS-enabled MICA2 motes collecting temperature, moisture content, and barometric pressure data. The data is communicated to a base station which records it in a database and provides services for different applications. The experiments show that most of the motes in the burned area were capable of reporting the passage of the flame before being burned. The critical point is that every node crossed by the fire is irreversibly destroyed. Another approach [4] follows a similar protocol by using the same type of device, but in addition, by considering the communication between the base station and the outside (through an Internet network). A Forest fire Surveillance System was designed for mountains in South Korea [5]. The authors provide a general structure for sensor networks and provide details for a forest fire detection application. The sensor types, operating system and routing protocol are discussed. Sensor nodes use a minimum cost path forwarding to send their readings to a sink which is connected to the Internet. The data is reported to a middleware which calculates the forest fire risk level according to formulas defined by forestry service. The calculation depends on daily measurement of relative humidity, precipitation, and solar radiation. The results are recorded in a database that can be accessed by web applications through the Internet.

Previous studies [2,3] show the capacity of the deployed WSN to detect a fire. However, after the passage of the fire, the network is partially destroyed and the data transmission cannot go on. The destroyed sensors can no longer serve, for instance to determine the direction of the fire. How to distinguish if the signal loss is a fire or a failure? It seems also difficult to envisage that after each fire it is necessary to have a campaign to replace all burned sensors. Finally, even if the previous works are a first approach, they show that the sensor destruction is a strong deficiency of the overall WSN when immerged in a fire. This must be upgraded. Finally, there is no available result demonstrating the ability of a WSN to track the kinematics of a fire i.e., the spatial and temporal movement of its reaction zone. This is indispensable in order to be able to estimate the rate of fire spread and the direction of fire propagation. These parameters are crucial not only in the rescue actions for fight during a fire but also for fire spread modeling.

### 1.2. Contributions and Paper Organization

The aim of this paper is to prove the ability of a WSN to monitor a fire by predicting, detecting and measuring the features of a fire. We know that the two steps are realizable with a WSN, but only by causing the irreversible destruction of the node by the fire. We introduce in this paper a major contribution by designing a special protection called Firesensorsock dedicated to the thermal insulation of the sensors leaving intact their ability to sense thermal data. Thus the sensor protected with Firesensorsock can resist the fire and the sensor can continue to transmit a data flow to the final user. This double system (WSN + Firesensorsock) is able to predict, detect and follow a fire and appears to be an efficient tool for firemen.

The paper is organized as follows. In Section 2, we introduce the materials used and methods of these series of experiments. Section 3 shows the results under different use configurations. In Section 4, an analysis and a criticism of the results are presented, and we conclude the paper in Section 5.

## 2. Materials and Methods

In these experiments we used two types of motes under different weather conditions. The Wireless Sensor Technologies used is provided by Crossbow Technology. We use two types of motes: MICA2 1^st^ generation and MICA2 3^rd^ generation [6].

The objective of this study is the performance evaluation of a WSN in a natural spreading fire. The works of Doolin [2] show that a WSN is able to detect a fire. However the authors confirm in the conclusion of their work that it would be interesting if a WSN could resist the fire. Indeed, we can observe in the previous mentioned work that several nodes were destroyed by the travelling fire. The first reason is the thermal impact of the fire on the hardware. The lifetime of the sensors in a fire is on the scale of seconds. The challenge of our work is the increase of the lifetime of the sensors up to several minutes for allowing a correct tracking of the fire. A short node lifetime blurs the tracking of the fire spread. This lifetime can be formally defined as the delay of a continuous data transmission. It can be measured as uninterrupted curves along the X-coordinate axis.

### 2.1. Wireless Sensor Technologies

The two types of sensors used on the different experiments are presented in the Table 1. The details of the different materials are available on the Crossbow Technology website [4]. The useful data collected during fire tests are temperature and relative moisture content.

We began the series of experiments with the 1^st^ generation of the MICA2 motes (equipped with a 400 MHz Multi-Channel Radio Transceiver). In order to make the system evolve, we used then the last generation type MICA2 sensor. These ones are different from the 1^st^ generation because they use a 868/916 MHz Multi-Channel Radio Transceiver. The MICA2 sensors are based on the TinyOS operating system, an Open Source operating system designed specifically for wireless sensor networks. It respects an architecture based on a combination of components, reducing the code size required for its implementation. This is in accordance with the memory constraints of sensor networks. All sensors use the Xmesh routing protocol (initially reliable route protocol). This routing protocol defines a cost for each node in the network according to its parents: this cost is defined by the quality of radio link.

For the data analysis, we used the graphical user interface GUI MOTEVIEW 1.0 and 2.0. MOTEVIEW provides a programming interface of the sensors but it also allows the real-time monitoring of a network operation and data extraction.

### 2.2. Firesensorsocks

**Figure 2.**
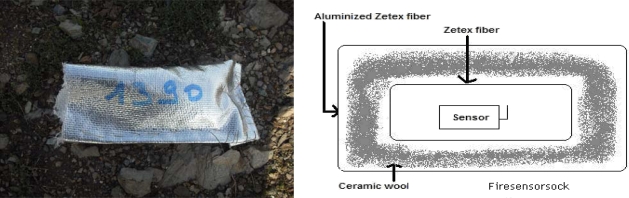
Firesensorsock.

In this subsection, we introduce a new device to protect the sensor under fire conditions. We have developed a way to thermally protect the sensors during the experiments, preventing their destruction and extending their lifetime beyond the fire event. With this thermal insulation, the network can be reused for subsequent events. The challenge is mainly to provide a protection able to preserve continuous flow of data from sensors embedded in a thermally destructive environment, namely a large scale natural fire. Our protection, called Firesensorsock, is presented in Figure 2. It should be noticed that, when sensors are covered with Firesensorsock the temperature and humidity measured during fire experiments are relative to the ones inside the protection. However, the sensing of any significant change of thermal data in the protection, due to the change of external thermal medium governed by the fire allows for fire detection and tracking.

The Firesensorsock consists in several layers of thermal insulation materials: a layer of simple Zetex fiber, a layer of ceramic wool and a final layer of aluminized Zetex fiber. These layers are fixed to one another with Kevlar thread. We thus achieve a level of protection allowing the sensor to support a prolonged fire contact and also wireless communications without any disturbance.

### 2.3. Weather Conditions and Equipments

The experiments have been performed under three different weather conditions, namely during summer 2007 and autumn 2008. In 2007, MICA2 motes sensors were used and we deployed only four units. A second campaign was performed in 2008, during which the number of sensors has been increased and the last generation of sensors has been used. These protocols are summarized in Table 2.

**Figure 3.**
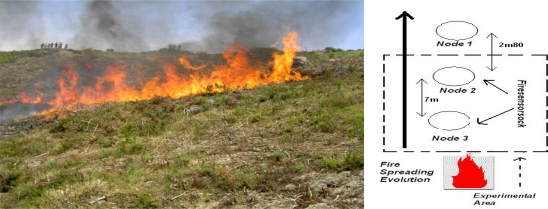
MICA2 1^st^ GEN Experimental Area.

**Figure 4.**
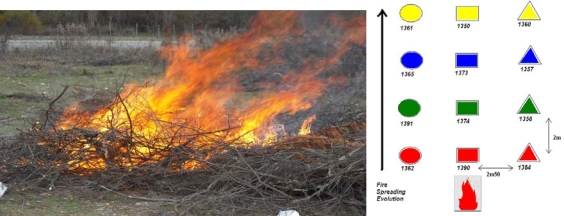
MICA 3^rd^ GEN Experimental Area with 12 sensors on a square with 2 m 50 in width and 2 m distance between the nodes.

**Figure 5.**
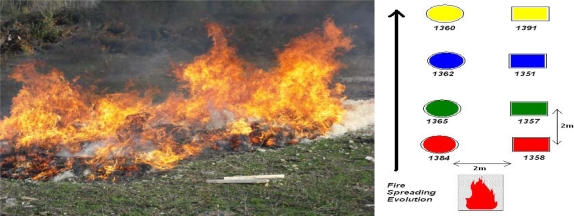
MICA 3^rd^ GEN Experimental Area with eight sensors on a square with 2 m in width and 2 m in distance between the nodes.

In Figures 3, 4 and 5 we show the different experimental areas with their different characteristics.

In the Figures 3, 4 and 5 we use different symbols to represent the line of sensors. In the fire spread direction, we use a special caption: circle, square or triangle. Perpendicularly, we use different colors to identify the different line of sensors. These captions are used in the following curves.

## 3. Results

### 3.1. WSN Performance with MICA2 1^st^ GEN Network

**Figure 6.**
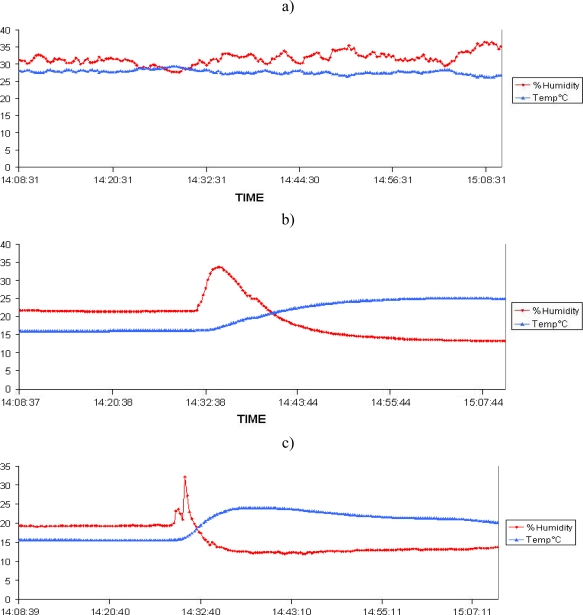
Results from a) Node 1; b) Node 2 and c) Node 3 showing that the nodes in a fire can transmit data continuously.

First, a fire test was conducted with four MICA2 1^st^ GEN sensors (Figure 3). Node 1 was the witness node without protection located outside the vegetation plot, the nodes 2,3 with Firesensorsock were located inside the area to be burnt. The temperature and humidity curves extracted from the database are presented in Figures 6a–c, which show the data from Node 1, Node 2 and Node 3, respectively

Firstly, we can observe that the lifetime of each node is preserved because we can clearly observe continuous data transmission in the different curves. The sensors resisted the temperature increase and remained operational, even after the passage of the fire. Node 1 (Figure 6a) is used for setting a reference temperature. This corresponds to the air temperature inside a Firesensorsock, close to the ambient air temperature. The data collected are sent to the base station and then transmitted to the laptop. The measurements collected with Nodes 2 and 3 are provided in Figures 6b and 6c. These curves present the time evolution of temperature and humidity in the Firesensorsock.

Secondly, with these curves, we are able to determine instants of the thermal impact of the fire on sensors. Then, from these temporal profiles, one can deduce the kinematic properties of a fire front during its spread, and in peculiar, the rate of fire spread. Indeed, one can observe in Figure 6(b),(c) that the flame impact on Node 2 coincides with two significant changes in both the temperature and humidity curves: the temperature curve rises exactly at the arrival of fire at the sensor location. It should be noticed that humidity also increases and this occurs slightly before the temperature rise. This can be related to the evaporation of natural moisture inside the thermal protection and surrounding vegetal fuel. Evaporation is the first step in the thermal degradation of solid materials and it always occurs ahead of the flame front. The nodes transmit their data periodically to the base station. These data are recorded with a time of receipt which never exceeds 5 s, whereas the shortest time scale of the fire front spread is about 250 seconds – namely the flame residence time at a given spatial position. This time scale guarantees that the response time of the WSN is appropriate to capture the faster movement of the overall fireline.

Thirdly, we can thus determine the rate of fire spread based on the temperature increase. We know that the Node 1 is spaced 9.8 m and 16.8 m apart from Nodes 2 and 3 respectively. The deduced rate of spread is about 4 cm·s^−1^. Such a value coincides with the one obtained if the rate of fire spread is computed by using the instant of humidity change. This value was finally confirmed by lateral video recordings. The agreement obtained about the ROS (rate of spread) between the WSN and the video recording is promising because it illustrates the ability of this sensor set to determine kinematic features of the fire in a given direction.

### 3.2. WSN Performance with 12 MICA2 3rd GEN

**Figure 7.**
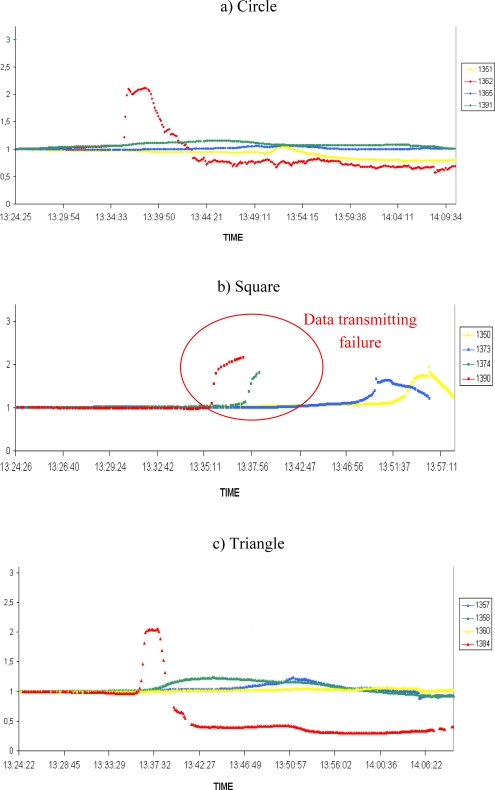
Humidity results of the 12 MICA2 3^rd^ GEN with a failure of nodes 1374 and 1390 without continuous data transmission.

**Figure 8.**
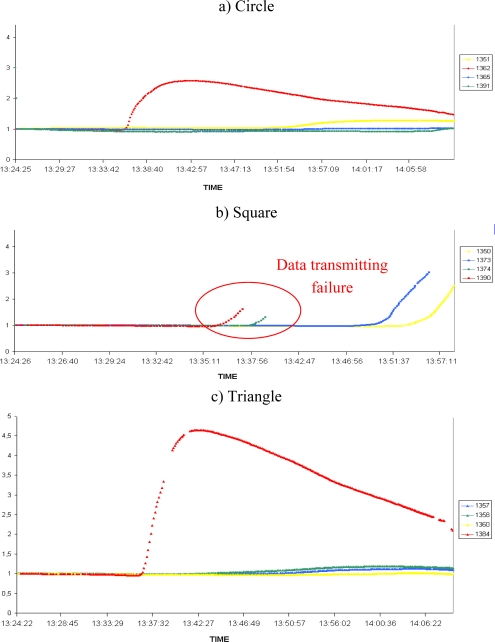
Temperature results of the 12 MICA2 3^rd^ GEN.

The previous results illustrate the potential efficiency of a protected WSN to gain valuable physical data during the spread of a natural fire. In a second campaign of experiments the number of nodes increased in order to display a grid all above the vegetation plot (Figure 4). The last generation of Crossbow motes, namely the MICA2 3^rd^ GEN was used. The results are shown in Figures 7a–c (humidity) and Figure 8a–c (temperature) on normalized curves. Figures 7a and 8a correspond to a circular line of sensors, 7b and 8b to a square line and 7c and 8c to a triangular line on the experimental plot. The colours are used to distinguish the lines perpendicular to the direction of the fire spread.

We can see in the Figures 7 and 8 different triggering of sensors, indicating the fire’s propagation. These allow us to track the spread of the fire. However, we can observe on the Figure 7 and Figure 8 some communication failures of several nodes (sensors 1350, 1373, 1373 and 1390). These problems are the results of failure in some of the protections, due to the destruction of some seams on the square line. This leads the corresponding nodes to saturate, causing an interruption of data sensing and transmission. This problem can be interpreted as a system failure already observed in the works of Doolin [2]. The lifetime of the sensors is clearly reduced and no longer unlimited.

In subsequent experiments, the protections were strengthened at the seams. These failures may have caused the malfunctioning of four sensors in the square line but the other eight sensors were efficiently protected. However, despite of these losses, we can observe that our system is able to detect and to track a fire when the integrity of the Firesensorsock is preserved. We can clearly distinguish on Figures 7 and 8 that the red nodes have detected the fire first (fire front at the beginning of the experiment) and just after, the circle line has detected it.

### 3.3. WSN Performance with 8 MICA2 3^rd^ GEN

**Figure 9.**
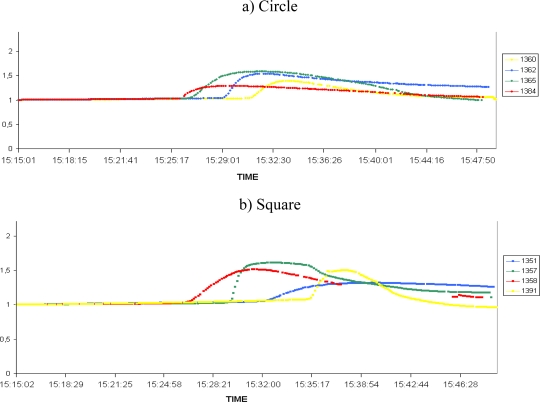
Humidity results of the 8 MICA2 3^rd^ GEN with a continuous data transmission.

**Figure 10.**
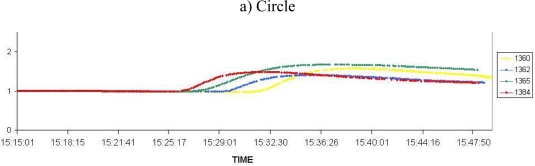

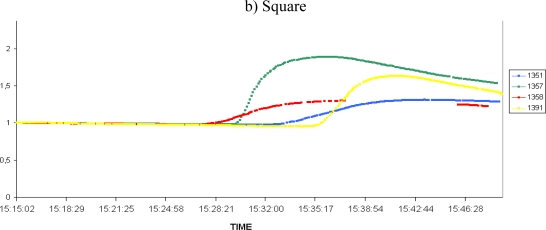
Temperature results of the eight MICA2 3^rd^ GEN sensors.

We show the results of eight sensors deployed as a grid in Figure 9 (humidity) and Figure 10 (temperature), according to the previously described caption. It is important to recall that for this experiment we have strengthened our Firesensorsocks so they can better resist the fire’s passage.

In these results we can see that the lifetime of the nodes is increased without limits. Indeed, the nodes resisted the fire and the data transmission was not limited by the impact of the fire on the nodes. This allows us to calculate exactly the fire’s spread. The lifetime is now only limited by the lifetime of the batteries and for the Crossbow Technology, whose lifespan is measured in years.

In both figures, all sensors are triggered by the arrival of the fire. We can distinguish exactly the direction of the fire’s spread. The estimation of the rate of fire spread was possible because no node is destroyed by the fire in these experiments: special caution has been exercised at each seam of the Firesensorsock. It should be noticed that the computed rate of spread is not the same over the whole grid. This feature is consistent with real fire dynamics: indeed, according to infrared video imaging of the horizontal plot, the fire front propagates with a strong asymmetry. This is consistent with the measurement performed with our WSN system which shows that during this experiment, the fire has moved faster on the line from the sensor 1384 to sensor 1360 and its rate of spread was 8 cm·s^−1^. Conversely, on the line from sensor 1358 to sensor 1391 the rate of spread was about 1.3 cm·s^−1^. The designed WSN system is therefore able to detect the different rate of fire spread along a single fire front. This proves that its sensitivity is able to take into account heterogeneities.

## 4. Discussion

Our contribution in this framework is to provide a new tool for monitoring wildland and forest fires using WSN according to three axes: sensing, detecting and tracking a wildfire. The technological challenge has been increasing the lifetime of the nodes in order to allow users able to calculate the rate of fire spread and determine the space and time evolution of the phenomenon. This is possible when nodes of a WSN are thermally protected, for instance by using the Firesensorsocks designed in this study.

### 4.1. Sensing

When convenient instruments are plugged in on each node, a Wireless Sensor Network is by definition able to sense environmental data. In wildfires, WSNs offers an efficient tool to provide critical environmental data: the previous results point out that the variations of hygrometry and temperature inside the shock can be measured by the sensor in a continuous flow of data. For the future, one can plan developing each node for measuring the incident heat flux which governs the time evolution of temperature and hygrometry inside the sock. A model for heat conduction through the sock and an inverse method must be derived for expressing incident fluxes as a function of temperature and hygrometry inside the sock, but if done, this would allow the upgrade of the WSN for the measurement of the spatial distributions of real heat fluxes emitted from the fire: such information is central in real-time fire safety strategies.

### 4.2. Detection

The sensing of the thermal data inside the sock which suddenly varies according to the presence of a strong thermal environment, i.e., a flame, leads to the opportunity to detect the presence of a fire. This point is very important for managing fire alerts in a specific area because it determines the action of the first fire rescue team. It is important to notice that the efficiency of the whole WSN strongly depends on the WSN response time regarding the faster time scale of the fire. This point must be emphasized because fire is a non linear transport phenomenon and a large range of space and time scales may coexist. In the present studies, the time scale of the fire propagation is about 250 s, so the system which detects significant variations of temperature and hygrometry at 0.2 Hz is adapted to the investigated dynamics, but it would probably need to be modified if used to monitor faster hazardous fire phenomena such as backdrafts.

### 4.3. Tracking

This last point is the overall objective of our contribution. In the previous work [2], it was impossible to track the fire despite of sensing and detecting capabilities of the sensors because the motes were destroyed by the fire. Our solution is to protect the mote using a thermal shield called Firesensorsock. The goal of this protection is to dampen the thermal impact from the fire on the motes during the fire spread and contact. This shield must also allow both a continuous emission of data and the temperature and the hygrometry inside the sock to vary on short time scales for locating the fire’s position. In this case, the tracking of the fire spread during its spatial and temporal evolution is possible with a spatial accuracy governed by the number of nodes and a temporal accuracy due to the WSN response time. As a summary, one can consider that:

1. the protected WSN goes on transmitting the data during the fire and allows an intrusive vision of the phenomenon; the flow of data is not interrupted and it is a reliable upgrade for WSNs.
2. the protected WSN allows one to calculate the fire spread, even if different rates of fire spread exist along the same fire line, by scanning the effect of fire heterogeneity.

It is important to insist on the following: the thermal data, i.e., temperature and hygrometry, detected by the protected mote are not the ones of the surrounding external environment. We rather demonstrate in this work that the role of our protection enhances the performance of a WSN when exposed to a large scale natural fire. The efficiency of the network facing to another kind of combustible generating others external conditions by combustion should also be investigated.

Finally, another feature of the network that should be studied concerns transmission. In multihop networks, the time of fire detection by a mote and the time of arrival on the base station is an important parameter which can modify the phenomenon perception in the time [7]. Indeed, we have not analyzed the radio transmission delay by varying the number of sensors under repeatable fire conditions. In the present work, the number of nodes is not important enough to present any effect on the data transmission delay, but this parameter must be taken into account if the number of nodes increases.

## 5. Conclusions

The objective of this paper was to analyse the effect of fire on a protected WSN. Because it is not possible to follow the evolution of a fire with the current systems, we tried to provide a new solution to protect the sensors. The double system (sensor + Firesensorsock) provides an adapted tool for natural fire scenarios. Results illustrate that the temperature and humidity variations in the socks allow us to relevantly determine the presence of a fire. If the response time is conveniently set up, i.e., sufficiently short in comparison to the shorter time scale of the fire, the WSN becomes a measurement system for the rate of fire spread in the open. As initially expected, the system responds accurately to the following requirements:

1. sensing thermal data in the open: the system is be able to accurately sense a gas temperature and an air moisture content;
2. detecting a fire: sudden rises in the time evolution of air temperature and humidity coincide with the contact of sensors with the fire;
3. tracking the fire spread during its spatial and temporal evolution: by relating the spatial position of the sensors in the network with the instant of rises in temperature and air humidity allows one to track the displacement of the fire front.

The different tests performed during these fire experiments illustrate the ability of our system to track the fire evolution. The problems encountered in the second set of experiments due to the destruction of seams have been solved by strengthening these seams and the system resulting from this upgrade was able to detect different rates of fire spread (Experiment 3) for different fire intensities. This point is a great step forward. Our thermal insulation system reduces failures and thus provides a sensor network which is resistant to prolonged contact with s fire. We also observe that data flows are not interrupted during the experiments. Firesensorsocks therefore allow data flow transmission by the protected system during the fire. This makes our system behave as a real robust and reusable intrusive monitoring tool, which respresents a real advance in the field.

For future work, this set of devices should be improved in order to measure the external heat fluxes impacting the nodes, instead of the temperature and hygrometry inside the thermal protection. This suggests that further tests must therefore be performed with this system. Finally, we also need to investigate the responses of larger networks and the effects of stronger fire intensities, i.e., finally performing experiments closer to real conditions.

## Figures and Tables

**Figure 1.**
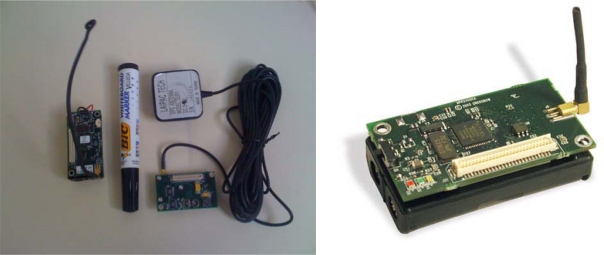
MICA2 and MTS420.

**Table 1.** Characteristics of motes and the equipments.

**Motes**	**Processor**	**Radio**	**Frequency**	**Sensorboard**	**Base Station**	**Data collected**
MICA2 1^st^ GEN	Atmel ATmega128L	MPR400	4OO MHZ	MTS420	MIB 510 (serial port)	Temperature, Moisture content, Pressure Light, GPS

MICA2 3^rd^ GEN	Atmel ATmega128L	MPR900	868/916 MHz	MTS400	MIB 520 (USB port)	Temperature, Moisture content, Pressure Light

**Table 2.** Weather conditions and equipment used.

**Weather**	**Number of sensors**	**Software**	**Type of sensors / BaseStation**	**Vegetation**
Sunny, hot	4	Motewiew 1.0	Mica2 1^st^ GEN, MIB 510	Dry plants
Wet, cold	12	Motewiew 2.0	Mica2 3^rd^ GEN, MIB 520	Wet plants
Wet, rainy, cold	8	Motewiew 2.0	Mica2 3^rd^ GEN, MIB 520	Wood chips
